# Single Nucleotide Polymorphisms in MiRNA Binding Sites of Nucleotide Excision Repair-Related Genes Predict Clinical Benefit of Oxaliplatin in FOLFOXIRI Plus Bevacizumab: Analysis of the TRIBE Trial

**DOI:** 10.3390/cancers12071742

**Published:** 2020-06-30

**Authors:** Mitsukuni Suenaga, Marta Schirripa, Shu Cao, Wu Zhang, Dongyun Yang, Chiara Cremolini, Sabina Murgioni, Sara Lonardi, Yan Ning, Satoshi Okazaki, Martin D. Berger, Yuji Miyamoto, Afsaneh Barzi, Fotios Loupakis, Alfredo Falcone, Heinz-Josef Lenz

**Affiliations:** 1 Division of Medical Oncology Norris Comprehensive Cancer Center, Keck School of Medicine, University of Southern California, 1441 Eastlake Avenue, Los Angeles, CA 90033, USA; martaschirripa@gmail.com (M.S.); Wu.Zhang@med.usc.edu (W.Z.); Yan.Ning@med.usc.edu (Y.N.); okazaki3332@gmail.com (S.O.); martindberger@gmail.com (M.D.B.); miyamotoyuji@gmail.com (Y.M.); afsaneh.barzi@myaccesshope.org (A.B.); LENZ@med.usc.edu (H.-J.L.); 2 Medical Oncology Unit 1, Department of Clinical and Experimental Oncology, Veneto Institute of Oncology, IOV-IRCCS, via Gattamelata 64, 35128 Padua, Italy; sabina.murgioni@ioveneto.it (S.M.); sara.lonardi@ioveneto.it (S.L.); fotiosloupakis@gmail.com (F.L.); 3 Department of Preventive Medicine, Norris Comprehensive Cancer Center, Keck School of Medicine, University of Southern California, 1441 Eastlake Avenue, Los Angeles, CA 90033, USA; caoshu1988@gmail.com (S.C.); donyang@coh.org (D.Y.); 4 Unit of Medical Oncology 2, Department of Translational Research and New Technologies in Medicine and Surgery, Azienda Ospedaliera Universitaria Pisana, University of Pisa, Via Roma 67, 56126 Pisa, Italy; chiaracremolini@gmail.com (C.C.); alfredo.falcone@med.unipi.it (A.F.)

**Keywords:** NER, oxaliplatin, metastatic colorectal cancer, RPA2

## Abstract

Background: The nucleotide excision repair (NER) pathway participates in platinum-induced DNA damage repair. Single nucleotide polymorphisms (SNPs) in miRNA-binding sites in the NER genes *RPA2* and *GTF2H1* are associated with the risk of colorectal cancer (CRC). Here, we analyzed whether *RPA2* and *GTF2H1* SNPs predict the efficacy of oxaliplatin in metastatic CRC (mCRC) patients. Patients and methods: Genomic DNA was extracted from blood samples from 457 patients with mCRC enrolled in the TRIBE trial, which compared first-line FOLFOXIRI plus bevacizumab (BEV) (*n* = 230, discovery cohort) and first-line FOLFIRI plus BEV (*n* = 227, control cohort). SNPs were analyzed by PCR-based direct sequencing. Results: In the FOLFOXIRI + BEV-treated cohort expressing wild-type *KRAS*, progression-free survival (PFS) was shorter for the *RPA2* rs7356 C/C variant subgroup than the any T allele subgroup in univariate analysis (9.1 versus 13.3 months respectively, hazard ratio (HR) 2.32, 95% confidence interval (CI): 1.07–5.03, *p* = 0.020) and this remained significant in multivariable analysis (HR 2.97, 95%CI: 1.27–6.94, *p* = 0.012). A similar trend was observed for overall survival. In contrast, patients expressing mutant *RAS* and *RPA2* rs7356 C/C variant had longer PFS with FOLFOXIRI + BEV than with FOLFIRI + BEV (12.1 versus 7.6 months, HR 0.23, 95%CI: 0.09–0.62, *p* = 0.002) but no superiority of FOLFOXIRI + BEV was observed for the *RAS* mutant, *RPA2* rs7356 any T variant subgroup (11.7 versus 9.6 months, HR 0.77, 95%CI: 0.56–1.07, *p* = 0.12) or the *RAS* wild-type, *RPA2* rs7356 C/C variant subgroup. Conclusion: *RPA2* SNPs may serve as predictive and prognostic markers of oxaliplatin responsiveness in a *RAS* status-dependent manner in mCRC patients receiving FOLFOXIRI + BEV.

## 1. Introduction

Nucleotide excision repair (NER) is an important component of the cellular DNA damage response (DDR) that removes toxic platinum–DNA adducts induced by oxaliplatin treatment [1]. NER-mediated removal of damaged DNA occurs through multiple steps: (i) recognition of DNA damage, (ii) assembly of protein complexes at the site of damage, (iii) excision of DNA, and (iv) synthesis and ligation of DNA for gap filling [2]. During NER, transcription factor IIH (TFIIH) and replication protein A (RPA) bind sequentially to damaged DNA to enable single-stranded DNA stabilization, and subunits of TFIIH, xeroderma pigmentosum, and complementation group B (XPB) and group D (XPD) helicases then unwind the DNA double helix at the damaged site. The endonuclease XPG and XPF-excision repair cross-complementing group 1 (ERCC1) complex then performs dual DNA incision followed by excision of the single-stranded DNA fragment. DNA ligase I then synthesizes DNA to fill the gap (Figure S1).

MicroRNAs (miRNA) are small, single-stranded, non-coding RNA molecules that interact with the 3′-untranslated region (3′UTR) of target mRNAs to regulate their stability and/or degradation. Such post-transcriptional regulation of gene expression by miRNAs plays crucial roles in controlling many physiological cellular processes as well as pathological events such as carcinogenesis [3,4]. A hospital-based case-control study by Landi et al. first identified significant associations between genetic polymorphisms in miRNA-binding sites in mRNAs and the risk of colorectal cancer (CRC) [5]. More recently, polymorphisms in the miRNA-binding sites *RPA2* and general transcription factor IIH subunit 1 (*GTF2H1*) mRNAs, both of which are involved in the NER pathway, have also been shown to correlate with CRC risk [6]. Many miRNAs are thought to affect the growth and chemotherapeutic response of cancer, as well as clinical outcomes, and are under investigation as potential targets for cancer therapy. However, whether polymorphisms in NER pathway-related genes affect the efficacy of oxaliplatin-based chemotherapies is still unclear.

The TRIBE trial (NCT00719797) compared the efficacy of FOLFOXIRI (5-fluorouracil (5-FU), leucovorin, oxaliplatin, and irinotecan) plus bevacizumab (BEV) and FOLFIRI (5-FU, leucovorin, and irinotecan) plus BEV in a first-line setting for patients with metastatic CRC (mCRC), and showed that the addition of oxaliplatin conferred significant progression-free survival (PFS) and overall survival (OS) benefits [7,8]. Given the previous findings showing a relationship between polymorphisms in NER pathway genes and CRC risk, we hypothesized that single nucleotide polymorphisms (SNPs) that affect miRNA binding to NER-related mRNAs may influence the repair of oxaliplatin-induced DNA damage, and thus serve as predictive or prognostic markers of oxaliplatin efficacy. To this end, we examined the relationship between SNPs in *RPA2* and *GTF2H1* in patients with mCRC in the TRIBE trial and determined their ability to predict the efficacy of FOLFOXIRI + BEV compared with FOLFIRI + BEV regimens.

## 2. Materials and Methods

### 2.1. Study Design and Patients

Two patient cohorts from the randomized phase III TRIBE trial [7] were investigated in this study: a discovery cohort (*n* = 230) treated with FOLFOXIRI + BEV and a control cohort (*n* = 227) treated with FOLFIRI + BEV in the first-line setting. The TRIBE study was a phase 3, randomized, open-label, multicenter trial conducted in 34 Italian centers with patients with unresectable mCRC who had not received chemotherapy or biologic therapy for their metastatic disease. From 17 July 2008 through 31 May 2011, a total of 508 patients were enrolled in the study: 256 patients were randomly assigned to FOLFIRI + BEV and 252 to FOLFOXIRI + BEV, and the primary endpoint of PFS was met [7]. In an updated analysis, the secondary endpoint of OS in the main cohort and treatment efficacy in *RAS* and *BRAF* molecular subgroups were assessed and FOLFOXIRI + BEV was confirmed as a valuable option for first-line treatment of mCRC patients regardless of *RAS* and *BRAF* status [8]. FOLFIRI + BEV (irinotecan 180 mg/m^2^, 5-FU bolus 400 mg/m^2^, 5-FU infusion 2400 mg/m^2^, leucovorin 200 mg/m^2^, BEV 5 mg/kg) or FOLFOXIRI + BEV (oxaliplatin 85 mg/m^2^, irinotecan 165 mg/m^2^, 5-FU infusion 3200 mg/m^2^, leucovorin 200 mg/m^2^, BEV 5 mg/kg) was administered every 2 weeks. Treatment was continued until disease progression, unmanageable toxicity, or patient refusal occurred. This study was approved by the Institutional Review Board of the University of Southern California for Medical Sciences. The protocol for this study was approved by the Institutional Review Boards of each participating site, and the molecular analyses were conducted at the University of Southern California/Norris Comprehensive Cancer Center in accordance with Good Clinical Practice Guidelines. The study was fully compliant with the Declaration of Helsinki and with the Reporting Recommendations for Tumor Marker Prognostic Studies (REMARK) guidelines.

### 2.2. Selection of Candidate SNPs

Candidate SNPs were selected according to the following criteria: (i) SNPs with biological significance according to the literature, (ii) tagging SNPs selected from HapMap genotype data with a r^2^ threshold of 0.8 (http://snpinfo.niehs.nih.gov/snpinfo/snptag.html), or (iii) SNPs with a minor allele frequency ≥ 10% in Caucasians according to the Ensembl Genome Browser (http://uswest.ensembl.org/index.html). Functional significance was predicted from the functional SNP (F-SNP) database [9] (Table S1). Potential gene functions were identified from public databases (https://www.ncbi.nlm.nih.gov, https://www.genecards.org/). In a comprehensive analysis of selected SNPs in the 3′UTR of NER genes by previous report, only *RPA2* rs7356 and *GTF2H1* rs4596 were associated with colorectal cancer risk when adjusted for the covariates, which contributed to our SNP selection [6]. Finally, SNPs of rs4596 in *GTF2H1* and rs7356 in *RPA2* were selected based on their previously reported association with CRC risk.

### 2.3. DNA Extraction and Genotyping

Genomic DNA was extracted from peripheral whole blood samples using QIAmp Kits (Qiagen, Valencia, CA, USA) according to the manufacturer’s protocol (www.qiagen.com). SNPs were analyzed using PCR-based direct DNA sequence analysis with an ABI 3100A Capillary Genetic Analyzer and Sequencing Scanner v1.0 (Applied Biosystems, Foster City, CA, USA). Forward and reverse primers are listed in Table S1. For quality control, 10% of the samples were randomly selected for direct DNA sequencing of the SNPs. The genotype concordance rate was ≥99%. The investigators who analyzed the SNPs were blinded to the clinical data.

### 2.4. Statistical Analysis

The primary endpoint of the current study was PFS and the secondary endpoints were OS and objective response rate (ORR). PFS was calculated from the date of randomization to the date of confirmed disease progression or death. OS was calculated from the date of randomization to the date of death from any cause. For surviving patients without disease progression, data were censored at the date of last follow up. For patients who were lost to follow up, data were censored at the date when the patient was last confirmed to be alive. The ORR was calculated as the proportion of patients who achieved complete response and partial response according to RECIST v1.0 criteria. Chi-square tests were used to examine the differences in baseline patient characteristics between two cohorts, associations between SNPs and clinical response were examined using Fisher’s exact test, and associations between SNPs and survival were estimated by the Kaplan–Meier method and log-rank test. The predictive or prognostic value of clinical factors and SNPs was identified by univariate analysis using codominant, dominant, or recessive genetic models as appropriate. Baseline characteristics significantly associated (*p* < 0.1) with PFS or OS were generally included in multivariable analysis using a Cox proportional hazards model. Subgroup analysis based on *KRAS* or *RAS* (*KRAS/NRAS*) mutational status was also performed. All analyses were carried out with SAS 9.4 (SAS Institute, Cary, NC, USA). All tests were 2-sided at a significance level of 0.050.

## 3. Results

### 3.1. Baseline Clinicopathological Characteristics of the Discovery and Control Cohorts

The median follow-up time, PFS, and OS were 46.6, 11.7, and 30.6 months respectively, for the discovery cohort (*n* = 230) and 49.0, 9.7, and 26.1 months respectively, for the control cohort (*n* = 227). The baseline clinicopathological characteristics of the patients are summarized in Table S2, and the associations between baseline characteristics and clinical outcomes are summarized in Tables S3 and S4.

Resection of the primary tumor was significantly associated with shorter PFS and OS in the discovery cohort, and higher ECOG, right-sided location, primary tumor resection, and mutant *BRAF* were significantly associated with shorter PFS and OS in the control cohort.

The frequencies of the genetic variants of the *GTF2H1* and *RPA2* SNPs in both cohorts satisfied the Hardy–Weinberg equilibrium (*p* > 0.01) using the exact test in Haploview software version 4.2.

### 3.2. Association between Clinical Outcome and SNPs in the Discovery Cohort

**Figure 1:**
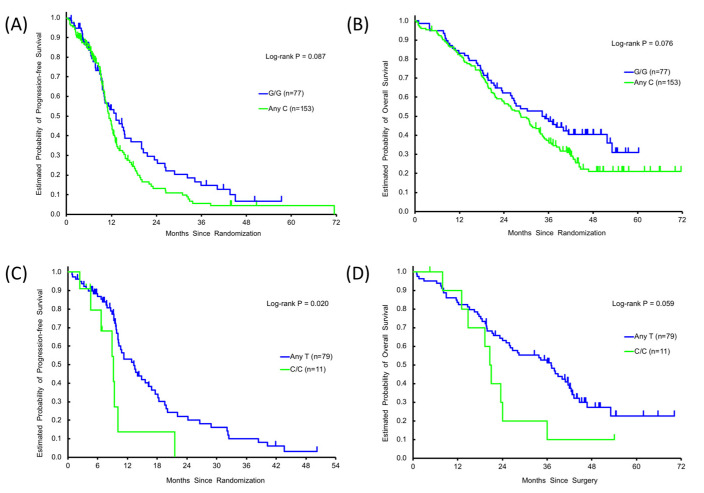
Single nucleotide polymorphisms (SNPs) and clinical outcomes. Progression-free survival (PFS) and/or overall survival (OS) by *GTF2H1* rs4596 variants, G/G (**―**) or any C (**―**) in the discovery cohort (**A,B**), and by *RPA2* rs7356 variant, C/C (**―**) or any T (**―**) in *KRAS* wild-type subgroup in the discovery cohort (**C,D**).

Table 1 shows the results of univariate and multivariate analyses of the associations between the SNPs and outcomes for patients in the FOLFOXIRI + BEV-treated discovery cohort and FOLFIRI + BEV-treated control cohort. Tumor response was not significantly associated with any specific *GTF2H1* or *RPA2* SNP variant in either cohort. Neither PFS or OS was significantly different among patient subgroups expressing *RPA2* rs7356 C/C or any T (T/T or T/C) allele. In contrast, patients with *GTF2H1* rs4596 G/C or C/C variants had shorter PFS and OS than patients with the *GTF2H1* G/G variant, although the differences did not reach the level of statistical significance (PFS: 11.3 versus 13.2 months, hazard ratio (HR) 1.32, 95% confidence interval (CI) 0.95–1.84, *p* = 0.087; OS: 28.6 versus 34.3 months, HR 1.36, 95%CI 0.97–1.92, *p* = 0.076). The trends in PFS and OS in the *GTF2H1* rs4596 G/G subgroup remained in the multivariable analysis (PFS: HR 1.37, *p* = 0.085; OS: HR 1.42, *p* = 0.066) (Table 1, Figure 1A,B).

Table 2 shows the associations between the SNPs and outcomes for patients expressing wild-type *KRAS* (*n* = 90) and wild-type *RAS* (*n* = 62) in the discovery cohort. Among the patients harboring wild-type *KRAS*, the *RPA2* rs7356 C/C variant was significantly associated with shorter PFS than the T/T or T/C variant (any T allele) subgroup (9.1 versus 13.3 months, HR 2.32, 95%CI 1.07–5.03, *p* = 0.020) and a similar trend, albeit not significant, was observed for OS (20.8 versus 37.1 months, HR 1.94, 95%CI 0.94–3.99, *p* = 0.059). In the *KRAS* wild-type subgroup, *GTF2H1* rs4596 any C variant was associated with shorter PFS than the G/G variant (10.8 versus 13.3 months, HR 1.64, 95%CI: 0.95–2.81, *p* = 0.045). In multivariable analysis, the detrimental effect of *RPA2* rs7356 C/C variant expression was significant for both PFS (HR 2.97, 95%CI 1.27–6.94, *p* = 0.012) and OS (HR 2.58, 95%CI 1.19–5.58, *p* = 0.016) (Figure 1C,D). Among patients harboring wild-type *RAS*, there were no significant differences in outcomes among patients expressing *GTF2H1* rs4596 variant alleles. However, the PFS was significantly shorter for patients with the *RPA2* rs7356 C/C variant compared with any T variant (HR 5.96, 95%CI 1.84–19.29, *p* = 0.003). In the discovery cohort, patients expressing mutant *KRAS* or *RAS* exhibited no significant SNP-associated differences in tumor progression or survival outcomes (Table 2 and Table S5).

### 3.3. Association between Clinical Outcome and SNPs in the Control Cohort

In the FOLFIRI + BEV-treated control cohort, there were no significant associations between the *GTF2H1* or *RPA2* SNPs and clinical outcomes in univariate analysis (Table 1). OS was significantly shorter in the *GTF2H1* rs4596 any C subgroup in multivariable (*p* = 0.038) but not univariate (*p* = 0.50) analysis. However, analysis of the *RAS* mutant subgroup of the control cohort (*n* = 115) showed that patients with the *RPA2* rs7356 C/C variant had significantly shorter PFS than those with any T allele in both univariate and multivariable analysis (7.6 versus 9.6 months, HR 1.95, 95%CI 1.03–3.72, *p* = 0.032 and HR 2.15, 95%CI 1.03–4.46, *p* = 0.040, respectively). In contrast, there were no significant *RPA2* or *GTF2H1* SNP allele-associated differences in outcomes among patients harboring wild-type *RAS* (Table S6).

### 3.4. Clinical Significance of RPA2 SNP Alleles and RAS Mutational Status in the FOLFOXIRI + BEV and FOLFIRI + BEV Cohorts

**Figure 2:**
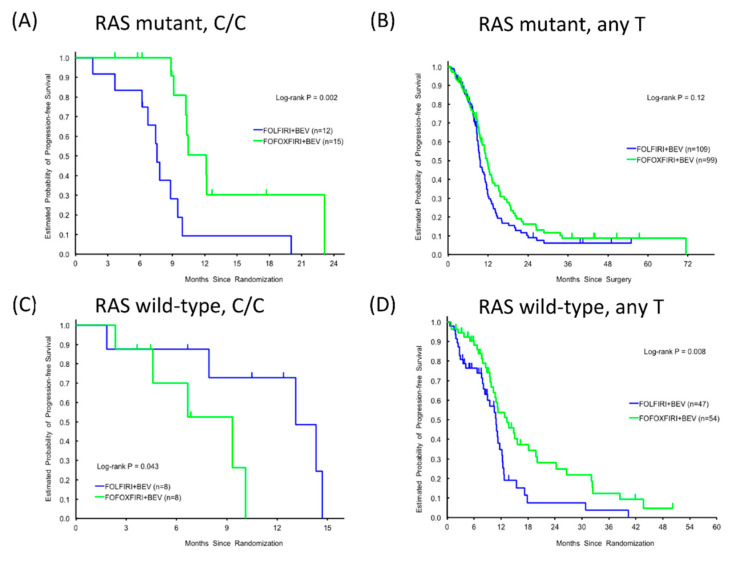
Progression-free survival (PFS) according to *RPA2* rs7356 variants, C/C or any T in the discovery cohort (**―**) and the control cohort (**―**): C/C, RAS mutant (**A**), Any T, RAS mutant (**B**), C/C, *RAS* wild-type (**C**), Any T, *RAS* wild-type (**D**).

Within the *RAS* mutant subgroup, patients expressing the C/C variant of *RPA2* rs7356 had a significantly longer PFS in response to FOLFOXIRI + BEV compared with FOLFIRI + BEV (12.1 versus 7.6 months, HR 0.23, 95%CI 0.09–0.62, *p* = 0.002, Figure 2A), whereas no significant treatment effect was detected for patients expressing *RPA2* rs7356 any T allele (11.7 versus 9.6 months, HR 0.77, 95%CI: 0.56–1.07, *p* = 0.12, Figure 2B). In contrast, patients expressing wild-type *RAS* and the RPA2 rs7356 *C/C* variant had shorter PFS in response to *FOLFOXIRI + BEV* than *FOLFIRI + BEV* (9.3 versus 13.1 months, HR 5.01, 95%CI: 0.92–27.01, *p* = 0.043, Figure 2C), whereas the opposite was true for patients expressing wild-type *RAS* and *RPA2* rs7356 any T allele (13.3 versus 10.8 months, HR 0.52, 95%CI: 0.32–0.85, *p* = 0.008, Figure 2D). Thus, the beneficial effect of oxaliplatin is influenced by both the *RAS* mutational status and the specific *RPA2* rs7356 allele.

## 4. Discussion

To our knowledge, our results represent the first evidence that SNPs in genes involved in NER of platinum-induced DNA damage are associated with the superior efficacy of FOLFOXIRI + BEV compared with FOLFIRI + BEV for patients with mCRC [7]. 

As one of the main DDR pathways for oxaliplatin-induced DNA damage, NER has been explored as a potential mechanism of resistance for oxaliplatin-based treatments [10]. Excision repair cross-complementing 1 (ERCC1) has also been implicated in oxaliplatin resistance [11,12,13] and intratumoral ERCC1 mRNA levels may be a potential marker of oxaliplatin-based chemotherapy resistance [11]. An in vitro study of two *KRAS* wild-type and two *KRAS* mutant CRC cell lines showed that ERCC1 was induced by oxaliplatin only in the *KRAS* wild-type cells, despite equivalent basal ERCC1 levels in all four cell lines. These data suggested that ERCC1 induction may contribute to oxaliplatin resistance in a *KRAS* status-dependent manner [12]. TFIIH and RPA2 are also important factors in NER [2,14], although whether their activity is influenced by *KRAS* status is unknown. Components of the NER process are known to physically and functionally interact with each other; for example, XPF–ERCC1 complexes can interact with XPA–RPA complexes and TFIIH [15,16]. Thus, it is likely that multiple components of the NER could be involved in the mechanism of oxaliplatin resistance.

Previous studies have demonstrated associations between polymorphisms in miRNA-binding sites of mRNAs involved in NER and the risk of mCRC [5,6], which prompted our focus in the present study on SNPs in the miRNA-binding sites of *GTF2H1* and *RPA2* mRNAs. The most interesting findings were that *GTF2H1* rs4596 and *RPA2* rs7356 variants significantly influenced PFS only in FOLFOXIRI + BEV-treated patients expressing wild-type *KRAS*, and not in FOLFOXIRI + BEV-treated patients with mutant *KRAS* or FOLFIRI + BEV-treated patients, regardless of *KRAS* status. In both earlier studies of SNPs as biomarkers of CRC risk [5,6], variations in thermodynamic measures (Gibbs free energy) were used to assess SNP effects on miRNA–mRNA binding. *GTF2H1* rs4596 variant G, which is associated with a decreased risk of CRC, was predicted to reduce binding of miR-518a-5p and miR-527, leading to elevated *GTF2H1* expression and NER pathway activity. In contrast, miR-3149 and miR-1183 were predicted to show increased binding to *RPA2* rs7356 variant G, thereby decreasing RPA2 expression and increasing the risk of CRC. The authors of these studies concluded that the SNP variants influenced miRNA-mediated regulation of *GTF2H1* and *RPA2* mRNA levels and thus DNA repair activity and the cancer risk [6]. 

In our study, the G/G variant in *GTF2H1* rs4596 correlated with a trend towards longer PFS and OS in both cohorts, suggesting that the potential role of *GTF2H1* rs4596 in DDR was unaffected by the addition of oxaliplatin to the regimen. In contrast, *RPA2* rs7356 appeared to have no prognostic or predictive value for the whole study population; however, its clinical significance varied when *KRAS* and *RAS* mutant versus wild-type subgroups were assessed. We assumed that patients expressing the *RPA2* rs7356 G (C) allele would benefit more from the addition of oxaliplatin than those expressing the A (T) allele due to reduced expression of *RPA2* [6]. Nevertheless, we found that FOLFOXIRI + BEV was beneficial for mutant *RAS*-expressing patients with either *RPA2* rs7356 allele, but the C/C variant was associated with a significantly shorter PFS than any T allele in response to FOLFIRI + BEV. This suggests that *RPA2* rs7356 is prognostic for FOLFIRI + BEV but not predictive for FOLFOXIRI plus BEV. In patients expressing wild-type *RAS*, however, the outcomes of patients with the *RPA2* rs7356 C/C variant were comparable to those of patients expressing with any T allele in the control cohort. Furthermore, compared with patients receiving FOLFIRI + BEV, the beneficial effect of adding oxaliplatin was evident in the patients expressing any T allele, but the opposite pattern was observed for the C/C variant. This suggests that *RPA2* rs7356 is predictive for FOLFOXIRI + BEV. Several proteins in the NER pathway have been known to interact directly [17,18,19,20]; for example, XPA is known to weakly interact with the ERCC1–XPF complex. Our results suggest that, in patients harboring wild-type RAS, the *RPA2* rs7356 C/C variant may interact with ERCC1 via XPA to promote its activity, resulting in resistance to oxaliplatin [12]. 

The strengths of the present study include the use of discovery and control cohorts from a randomized phase III trial to identify the predictive and prognostic value of oxaliplatin in mCRC patients. There are also several limitations to this study. Ideally, SNPs within all NER genes should be tested; however, we selected two candidate SNPs within two genes that showed a strong impact on colorectal cancer risk in a previous report [6] due to limited samples and cost. Further validation studies and preclinical investigations will be necessary to determine how SNPs in NER genes affect protein–protein interactions in the repair pathways of oxaliplatin-induced DNA damage.

## 5. Conclusions

In conclusion, the results of this study suggest that SNPs in the miRNA-binding sites of NER gene transcripts may affect the DDR to oxaliplatin-containing therapies. While *GTF2H1* rs4596 expression correlates with prognosis, *RPA2* rs7356 may serve as a *RAS* status-dependent prognostic and predictive marker of FOLFOXIRI + BEV sensitivity in mCRC patients. Further comprehensive analysis of SNPs within NER genes is warranted using another cohort receiving oxaliplatin-based chemotherapy.

## Figures and Tables

**Table 1:** Association between gene polymorphism and clinical outcome.

SNPs	Patients	Tumor Response			Progression-Free Survival					Overall Survival				
Variants	*N*	CR + PR	SD + PD	*p* Value *	Median, Months (95%CI)	HR (95%CI) †	*p* Value *	HR (95%CI) ‡	*p* Value *	Median, Months (95%CI)	HR (95%CI) †	*p* Value *	HR (95%CI) ‡	*p* Value *
**Discovery cohort**														
GTF2H1 rs4596				0.21			0.21		0.18			0.20		0.15
G/G	77	56(73.7%)	20(26.3%)		13.2(9.9,17.2)	Reference		Reference		34.3(25.8,51.8)	Reference		Reference	
G/C	117	74(66.7%)	37(33.3%)		1.3(10.1,12.2)	1.30(0.92,1.83)		1.32(0.90,1.93)		30.2(24.0,33.4)	1.38(0.97,1.97)		1.37(0.93,2.04)	
C/C	36	20(57.1%)	15(42.9%)		12.4(9.7,13.7)	1.41(0.87,2.28)		1.54(0.93,2.56)		26.7(17.9,39.1)	1.30(0.80,2.10)		1.59(0.95,2.66)	
				0.16			0.087		0.085			0.076		0.066
G/G	77	56(73.7%)	20(26.3%)		13.2(9.9,17.2)	Reference		Reference		34.3(25.8,51.8)	Reference		Reference	
AnyC	153	94(64.4%)	52(35.6%)		11.3(10.3,12.5)	1.32(0.95,1.84)		1.37(0.96,1.96)		28.6(23.4,33.4)	1.36(0.97,1.92)		1.42(0.98,2.07)	
RPA rs7356				0.25			0.47		0.31			0.60		0.92
T/T	87	60(71.4%)	24(28.6%)		11.2(9.9,14.1)	Reference		Reference		28.4(21.5,34.7)	Reference		Reference	
T/C	111	66(62.3%)	40(37.7%)		12.5(10.9,13.7)	1.22(0.87,1.72)		1.29(0.90,1.84)		33.4(25.2,37.1)	1.18(0.84,1.66)		1.08(0.76,1.53)	
C/C	32	24(75.0%)	8(25.0%)		10.3(9.1,21.6)	1.20(0.72,1.98)		1.37(0.80,2.34)		30.9(19.3,36.8)	1.05(0.64,1.74)		1.03(0.61,1.73)	
				0.33			0.76		0.48			0.83		0.96
AnyT	198	126(66.3%)	64(33.7%)		12.0(10.8,13.2)	Reference		Reference		30.2(25.9,34.4)	Reference		Reference	
C/C	32	24(75.0%)	8(25.0%)		10.3(9.1,21.6)	1.07(0.68,1.70)		1.20(0.73,1.96)		30.9(19.3,36.8)	0.95(0.60,1.51)		0.99(0.61,1.60)	
**Control cohort**														
GTF2H1 rs4596				0.90			0.59		0.27			0.60		0.077
G/G	67	38(56.7%)	29(43.3%)		10.4(9.0,12.6)	Reference		Reference		26.3(20.5,36.1)	Reference		Reference	
G/C	110	64(59.8%)	43(40.2%)		9.7(8.6,10.8)	1.17(0.83,1.66)		1.34(0.93,1.94)		25.6(20.8,30.8)	1.07(0.76,1.52)		1.39(0.97,2.01)	
C/C	48	25(56.8%)	19(43.2%)		9.4(7.9,11.2)	1.21(0.79,1.85)		1.31(0.82,2.11)		24.3(17.8,31.6)	1.24(0.81,1.89)		1.67(1.05,2.66)	
				0.76			0.31		0.11			0.50		0.038
G/G	67	38(56.7%)	29(43.3%)		10.4(9.0,12.6)	Reference		Reference		26.3(20.5,36.1)	Reference		Reference	
AnyC	158	89(58.9%)	62(41.1%)		9.5(8.7,10.8)	1.18(0.85,1.64)		1.33(0.94,1.89)		25.1(21.1,29.1)	1.12(0.81,1.55)		1.45(1.02,2.07)	
RPA rs7356				0.45			0.38		0.61			0.55		0.77
T/T	89	49(57.0%)	37(43.0%)		10.3(9.2,11.6)	Reference		Reference		26.3(22.0,34.4)	Reference		Reference	
T/C	113	67(61.5%)	42(38.5%)		9.5(8.7,11.1)	1.18(0.86,1.64)		1.18(0.84,1.66)		26.2(21.1,32.5)	0.97(0.71,1.33)		1.01(0.72,1.40)	
C/C	25	12(48.0%)	13(52.0%)		8.8(7.5,10.8)	1.36(0.83,2.23)		1.16(0.70,1.95)		18.8(14.4,27.9)	1.26(0.77,2.08)		1.20(0.71,2.02)	
				0.27			0.35		0.80			0.28		0.47
AnyT	202	116(59.5%)	79(40.5%)		9.7(9.3,11.0)	Reference		Reference		26.3(23.0,31.3)	Reference		Reference	
C/C	25	12(48.0%)	13(52.0%)		8.8(7.5,10.8)	1.24(0.78,1.96)		1.06(0.66,1.71)		18.8(14.4,27.9)	1.29(0.81,2.06)		1.20(0.73,1.95)	

CR, complete response; PR, partial response; SD, stable disease; PD, progressive disease. *p* values < 0.05 were shown in bold. * *p*-value was based on the Fisher’s exact test for response, log-rank test in the univariate analysis (†) and Wald test in the multivariable analysis within Cox regression model (‡) adjusted for sex, age, ECOG performance status, primary tumor site, number of metastases, primary tumor resected, adjuvant chemotherapy, RAS status, and BRAF status.

**Table 2:** Subgroup analysis of *KRAS* wild-type and *RAS* wild-type in the discovery cohort.

SNPs	Patients	Tumor Response			Progression-Free Survival					Overall Survival				
Variants	*N*	CR + PR	SD + PD	*p* Value *	Median, Months (95%CI)	HR (95%CI) †	*p* Value *	HR (95%CI) ‡	*p* Value *	Median, Months (95%CI)	HR (95%CI) †	*p* Value *	HR (95%CI) ‡	*p* Value *
***KRAS wild-type***														
GTF2H1 rs4596				0.95			0.13		0.30			0.56		0.88
G/G	32	21(67.7%)	10(32.3%)		13.3(9.3,24.2)	Reference		Reference		37.1(19.7,NE)	Reference		Reference	
G/C	47	30(65.2%)	16(34.8%)		10.7(9.5,14.1)	1.60(0.91,2.82)		1.69(0.85,3.36)		33.8(21.0,40.9)	1.34(0.76,2.34)		1.15(0.62,2.17)	
C/C	11	7(70.0%)	3(30.0%)		13.7(3.7,18.4)	1.77(0.79,3.99)		1.60(0.66,3.89)		23.3(6.4,NE)	1.36(0.60,3.11)		1.20(0.49,2.94)	
				0.87			0.045		0.12			0.28		0.63
G/G	32	21(67.7%)	10(32.3%)		13.3(9.3,24.2)	Reference		Reference		37.1(19.7,NE)	Reference		Reference	
AnyC	58	37(66.1%)	19(33.9%)		10.8(9.7,14.1)	1.64(0.95,2.81)		1.66(0.87,3.17)		28.5(21.0,40.9)	1.34(0.78,2.30)		1.16(0.63,2.13)	
RPA rs7356				0.48			0.061		0.042			0.13		0.056
T/T	38	26(72.2%)	10(27.8%)		11.2(10.1,18.2)	Reference		Reference		28.1(21.2,NE)	Reference		Reference	
T/C	41	24(60.0%)	16(40.0%)		13.7(9.6,16.9)	1.12(0.66,1.92)		0.93(0.50,1.71)		38.0(24.1,42.5)	1.24(0.72,2.14)		0.99(0.54,1.82)	
C/C	11	8(72.7%)	3(27.3%)		9.1(4.6,10.1)	2.47(1.08,5.65)		2.85(1.16,7.03)		20.8(7.9,24.0)	2.19(1.00,4.80)		2.56(1.13,5.82)	
				0.65			0.020		0.012			0.059		0.016
AnyT	79	50(65.8%)	26(34.2%)		13.3(10.2,16.9)	Reference		Reference		37.1(25.9,42.0)	Reference		37.1(25.9,42.0)	
C/C	11	8(72.7%)	3(27.3%)		9.1(4.6,10.1)	2.32(1.07,5.03)		2.97(1.27,6.94)		20.8(7.9,24.0)	1.94(0.94,3.99)		20.8(7.9,24.0)	
***RAS wild-type***														
GTF2H1 rs4596				0.89			0.32		0.26			0.39		0.62
G/G	22	14(66.7%)	7(33.3%)		14.8(8.9,24.2)	Reference		Reference		37.1(19.4,NE)	Reference		Reference	
G/C	34	20(60.6%)	13(39.4%)		10.8(8.6,18.2)	1.50(0.77,2.95)		1.87(0.77,4.57)		34.3(21.0,42.5)	1.39(0.71,2.73)		1.12(0.50,2.48)	
C/C	6	4(66.7%)	2(33.3%)		13.7(3.7,19.8)	1.86(0.60,5.79)		2.44(0.66,8.97)		44.6(6.4,NE)	0.71(0.20,2.47)		0.58(0.15,2.25)	
				0.69			0.15		0.11			0.49		0.97
G/G	22	14(66.7%)	7(33.3%)		14.8(8.9,24.2)	Reference		14.8(8.9,24.2)		37.1(19.4,NE)	Reference		Reference	
AnyC	40	24(61.5%)	15(38.5%)		10.8(9.3,15.0)	1.55(0.81,2.98)		10.8(9.3,15.0)		35.9(21.0,42.6)	1.26(0.65,2.44)		0.98(0.46,2.11)	
RPA rs7356				0.59			0.015		0.008			0.18		0.22
T/T	25	16(66.7%)	8(33.3%)		10.8(8.9,20.0)	Reference		Reference		41.7(21.2,NE)	Reference		Reference	
T/C	29	16(57.1%)	12(42.9%)		13.7(9.6,24.2)	1.09(0.56,2.12)		0.62(0.25,1.53)		38.0(24.1,43.2)	1.40(0.70,2.78)		0.85(0.39,1.85)	
C/C	8	6(75.0%)	2(25.0%)		9.3(2.4,10.1)	3.70(1.18,11.56)		4.63(1.33,16.14)		20.6(7.9,23.6)	2.44(0.91,6.54)		2.16(0.73,6.35)	
				0.46			0.004		0.003			0.10		0.090
AnyT	54	32(61.5%)	20(38.5%)		13.3(10.3,18.2)	Reference		Reference		38.0(26.1,43.2)	Reference		Reference	
C/C	8	6(75.0%)	2(25.0%)		9.3(2.4,10.1)	3.51(1.20,10.29)		5.96(1.84,19.29)		20.6(7.9,23.6)	2.01(0.83,4.88)		2.36(0.88,6.39)	

CR, complete response; PR, partial response; SD, stable disease; PD, progressive disease. *p*-values < 0.05 are shown in bold. * *p*-value was based on the Fisher’s exact test for response, log-rank test in the univariate analysis (†) and Wald test in the multivariable analysis within Cox regression model (‡) adjusted for sex, age, ECOG performance status, primary tumor site, number of metastases, primary tumor resected, adjuvant chemotherapy, RAS status, and BRAF status. NE: estimates were not reached yet.

## Supplementary Materials

The following are available online at https://www.mdpi.com/2072-6694/12/7/1742/s1, Figure S1: Scheme of NER pathway, Table S1: Candidate SNPs of *GTF2H1* and *RPA2*, Table S2: Baseline patients and tumor characteristics, Table S3: Association of baseline characteristics with clinical outcome in the discovery cohort, Table S4: Association of baseline characteristics with clinical outcome in the control cohort, Table S5: Subgroup analysis of *KRAS* mutant and *RAS* mutant in the discovery cohort, Table S6: Subgroup analysis by *KRAS* and *RAS* status in the control cohort.
