# Supplementary material for: Single Nucleotide Polymorphisms in MiRNA Binding Sites of Nucleotide Excision Repair-Related Genes Predict Clinical Benefit of Oxaliplatin in FOLFOXIRI Plus Bevacizumab: Analysis of the TRIBE Trial
Source: Cancers (Basel). 2020 Jun 30;12(7):1742. doi: 10.3390/cancers12071742 (PMC7408001; doi:10.3390/cancers12071742)
Supplement: Supplementary file 1 [file cancers-12-01742-s001.pdf]

cancers

MDPI

Article

Single Nucleotide Polymorphisms in MiRNA Binding Sites of Nucleotide Excision Repair-Related Genes Predict Clinical Benefit of Oxaliplatin in FOLFOXIRI Plus Bevacizumab: Analysis of the TRIBE Trial

Mitsukuni Suenaga [1,*], Marta Schirripa [1,2], Shu Cao [3], Wu Zhang [1], Dongyun Yang [3], Chiara Cremolini [4], Sabina Murgioni [2], Sara Lonardi [2], Yan Ning [1], Satoshi Okazaki [1], Martin D. Berger [1], Yuji Miyamoto [1], Afsaneh Barzi [1], Fotios Loupakis [2], Alfredo Falcone [4] and Heinz-Josef Lenz [1]

1 Division of Medical Oncology Norris Comprehensive Cancer Center, Keck School of Medicine, University of Southern California, 1441 Eastlake Avenue, Los Angeles, CA 90033, USA; martaschirripa@gmail.com (M.S.); Wu.Zhang@med.usc.edu (W.Z.); Yan.Ning@med.usc.edu (Y.N.); okazaki3332@gmail.com (S.O.); okazaki3332@gmail.com (M.D.B.); miyamotoyuji@gmail.com (Y.M.); afsaneh.barzi@myaccesshope.org (A.B.); LENZ@med.usc.edu (H.-J.L.)

2 Medical Oncology Unit 1, Department of Clinical and Experimental Oncology, Veneto Institute of Oncology, IOV-IRCCS, via Gattamelata 64, 35128 Padua, Italy; sabina.murgioni@ioveneto.it (S.M.); sara.lonardi@ioveneto.it (S.L.); fotiosloupakis@gmail.com (F.L.)

3 Department of Preventive Medicine, Norris Comprehensive Cancer Center, Keck School of Medicine, University of Southern California, 1441 Eastlake Avenue, Los Angeles, CA 90033, USA; caoshu1988@gmail.com (S.C); donyang@coh.org (D.Y.)

4 Unit of Medical Oncology 2, Department of Translational Research and New Technologies in Medicine and Surgery, Azienda Ospedaliera Universitaria Pisana, University of Pisa, Via Roma 67, 56126 Pisa, Italy; chiaracremolini@gmail.com (C.C.); alfredo.falcone@med.unipi.it (A.F.)

* Correspondence: m.suenaga1972@gmail.com; Tel.: +1-(323)-865-3967; Fax: +1-(323)-865-0061

Supplementary

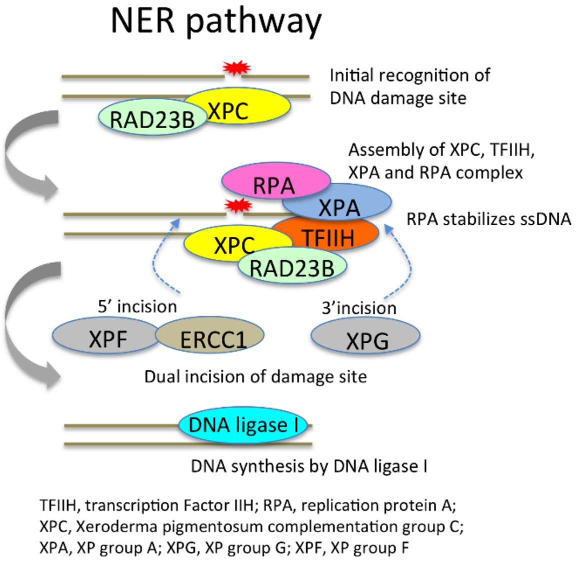

Figure S1. Scheme of NER pathway.

cancers

MDPI

Table S1. Candidate SNPs of *GTF2H1* and *RPA2*.

Genes<br>rs number	Allele location	Base exchange	MAF† (CEU)	Function of polymorphism	Forward / Reverse primer (5′-3′)
GTF2H1<br>rs4596	3′ prime UTR<br>Chromosome 17:35879999	G>C	0.45	Transcriptional regulation, conserved	F: TTGGTTCTGTGTTTCCTACGG<br>R: CAGCGCCATTGTCTTCTGAC
RPA2<br>rs7356	3' prime UTR<br>Chromosome 3:46370768	T>C	0.48	Transcriptional regulation	F: CACTGCTCAGAAAGAAAAACGA<br>R: TGAACATGAAGTTCTGCGGTA

MAF, Minor allele frequency; CEU, Caucasian; F. forward primer; R, reverse primer; Tag SNP, tagging SNP. † In Caucasians from the Ensembl Genome Browser: http://uswest.ensembl.org/index.html.

cancers

MDPI

Table S2. Baseline patients and tumor characteristics.

Cohort	Discovery cohort (*N*=230) TRIBE, FOLFOXIRI+BEV arm		Control cohort (*N*=227) TRIBE, FOLFIRI+BEV arm		*P* value *
	N	%	*N*	%	
Sex					
Male	137	60	137	60	0.86
Female	93	40	90	40	
Age (year)					
Median (range)	60 (29–75)		60 (29–75)		0.58
≤ 65	156	68	163	72	
> 65	74	32	64	28	
ECOG Performance status					
ECOG 0	205	89	187	82	0.050
ECOG 1-2	25	11	39	17	
Primary tumor site					
Right	78	34	56	25	0.039
Left	141	61	156	69	
Unknown	11	5	15	7	
Liver metastasis					
Yes	80	35	72	32	0.49
No	150	65	155	68	
Number of metastases					
1	100	43	99	44	1.00
2	91	40	89	39	
≥3	39	17	39	17	
Primary tumor resected					
Yes	157	68	143	63	0.24
No	73	32	157	69	
Adjuvant history					
Yes	29	13	28	12	0.93
No	201	87	199	88	
KRAS status					
Wild-type	90	39	96	42	0.44
Mutant	101	44	92	41	
Unknown	39	17	39	17	
RAS status					
Wild-type	62	27	55	24	0.57
Mutant	114	50	115	51	
Unknown	54	23	57	25	
BRAF status					

Wild-type	176	77	176	78	0.58
Mutant	15	7	12	5	
Unknown	39	17	39	17	

* *p* value was based on Chi-square test, or the Wilcoxon Rank Sums test when appropriate. Unknown groups were not included in the analysis.

Table S3. Association of baseline characteristics with clinical outcome in the discovery cohort.

		**Progression-free survival**			**Overall survival**		
	**N**	**Median (95%CI), months**	**Univariate HR (95%CI) †**	***P* value***	**Median (95%CI), months**	**Univariate HR (95%CI) †**	***P* value***
Sex				0.79			0.50
Male	137	12.0 (10.2, 13.2)	1 (Reference)		30.2 (23.4, 35.9)	1 (Reference)	
Female	93	11.3 (9.9, 14.0)	0.96 (0.70, 1.31)		30.9 (25.0, 35.4)	1.11 (0.82, 1.52)	
Age (year)				0.62			0.20
≤ 65	156	11.6 (10.2, 13.7)	1 (Reference)		30.8 (25.8, 37.2)	1 (Reference)	
> 65	74	11.7 (9.8, 13.3)	1.08 (0.78, 1.51)		27.9 (20.5, 33.5)	1.23 (0.89, 1.71)	
ECOG Performance status				0.11			**0.006**
ECOG 0	205	12.1 (10.7, 13.2)	1 (Reference)		30.9 (26.9, 35.9)	1 (Reference)	
ECOG 1-2	25	9.9 (4.2, 15.6)	1.45 (0.91, 2.29)		20.5 (8.9, 28.1)	1.88 (1.18, 2.98)	
Primary tumor site				0.50			**0.030**
Right	78	10.9 (8.8, 12.5)	1 (Reference)		25.5 (19.5, 30.8)	1 (Reference)	
Left	141	12.2 (10.8, 13.4)	0.89 (0.64, 1.24)		34.2 (28.1, 38.9)	0.70 (0.50, 0.97)	
Liver metastasis				0.53			0.47
Yes	80	11.1 (9.9, 13.7)	1 (Reference)		32.2 (24.1, 39.0)	1 (Reference)	
No	150	12.0 (10.3, 13.2)	0.90 (0.64, 1.26)		28.6 (23.4, 34.4)	1.13 (0.81, 1.56)	
Number of metastases				0.068			**0.007**
1	100	11.6 (10.1, 13.7)	1 (Reference)		34.3 (26.7, 42.5)	1 (Reference)	
2	91	13.0 (10.4, 15.0)	0.92 (0.65, 1.31)		30.8 (23.3, 36.0)	1.33 (0.94, 1.88)	
≥3	39	10.1 (7.8, 11.9)	1.47 (0.97, 2.22)		21.6 (13.1, 25.8)	1.96 (1.27, 3.02)	
Primary tumor resected				**0.005**			**0.024**
Yes	157	12.4 (10.9, 13.7)	1 (Reference)		33.5 (28.1, 38.2)	1 (Reference)	
No	73	10.1 (9.4, 12.4)	1.58 (1.12, 2.23)		23.4 (18.7, 30.6)	1.45 (1.05, 2.00)	
Adjuvant history				0.93			0.12
Yes	29	13.3 (12.1, 17.3)	1 (Reference)		38.0 (25.9, 61.8)	1 (Reference)	
No	201	11.2 (10.1, 12.4)	1.02 (0.66, 1.58)		28.5 (24.0, 33.8)	1.49 (0.89, 2.51)	
KRAS status				0.98			0.42
Wild-type	90	11.3 (10.1, 15.6)	1 (Reference)		34.3 (23.6, 40.0)	1 (Reference)	
Mutant	101	11.9 (10.3, 13.0)	1.01 (0.71, 1.42)		28.5 (21.7, 31.7)	1.15 (0.82, 1.61)	
RAS status				0.73			0.19
Wild-type	62	11.3 (9.6, 15.6)	1 (Reference)		37.1 (23.6, 42.6)	1 (Reference)	
Mutant	114	11.7 (10.3, 13.0)	1.07 (0.73, 1.56)		28.1 (21.7, 31.7)	1.28 (0.88, 1.86)	
BRAF status				0.092			**0.005**
Wild-type	176	12.0 (10.4, 13.4)	1 (Reference)		30.9 (25.8, 35.9)	1 (Reference)	
Mutant	15	7.9 (2.4, 15.0)	1.68 (0.90, 3.15)		14.6 (7.9, 26.1)	2.18 (1.25, 3.80)	

p values < 0.05 were shown in bold. * *p* value was based on the log-rank test for PFS and OS in the univariate analysis (†)

cancers

MDPI

Table S4. Association of baseline characteristics with clinical outcome in the control cohort.

		Progression-free survival			Overall survival		
	N	Median (95%CI), months	Univariate HR (95%CI) †	*P* value*	Median (95%CI), months	Univariate HR (95%CI) †	*P* value*
Sex				0.35			0.41
Male	137	9.7(8.8,10.8)	1 (Reference)		26.1(21.1,31.3)	1 (Reference)	
Female	90	9.5(8.6,11.1)	0.87(0.64,1.18)		25.4(20.8,31.6)	0.88(0.65,1.20)	
Age (year)				0.48			**0.012**
≤ 65	163	9.5(8.7,10.8)	1 (Reference)		27.9(23.9,33.9)	1 (Reference)	
> 65	64	10.3(9.0,11.9)	1.12(0.81,1.55)		21.3(18.6,26.1)	1.50(1.09,2.06)	
ECOG Performance status				**0.005**			**<0.001**
ECOG 0	187	10.3(9.4,11.5)	1 (Reference)		29.1(25.1,33.1)	1 (Reference)	
ECOG 1-2	39	8.7(5.7,10.3)	1.68(1.16,2.44)		14.6(7.7,18.4)	2.40(1.64,3.51)	
Primary tumor site				**0.036**			**<0.001**
Right	56	8.8(7.1,10.3)	1 (Reference)		19.9(14.7,23.9)	1 (Reference)	
Left	156	10.6(9.4,11.3)	0.70(0.50,0.98)		31.4(26.1,36.3)	0.51(0.36,0.73)	
Liver metastasis				0.52			0.24
Yes	72	10.3(8.3,11.9)	1 (Reference)		29.1(22.7,34.4)	1 (Reference)	
No	155	9.5(9.0,10.6)	1.12(0.79,1.57)		25.0(20.6,27.9)	1.21(0.88,1.67)	
Number of metastases				0.56			**0.022**
1	99	10.3(9.0,11.7)	1 (Reference)		30.2(25.4,35.8)	1 (Reference)	
2	89	9.5(8.6,11.1)	1.02(0.73,1.43)		25.0(20.6,31.3)	1.25(0.90,1.74)	
≥3	39	9.2(7.8,11.1)	1.23(0.82,1.85)		20.0(12.9,26.3)	1.76(1.16,2.65)	
Primary tumor resected				**0.003**			**<0.001**
Yes	143	10.8(9.5,11.6)	1 (Reference)		30.2(24.8,36.1)	1 (Reference)	
No	157	9.0(7.9,9.5)	1.58(1.15,2.16)		21.4(17.3,26.3)	1.67(1.23,2.27)	
Adjuvant history				0.076			**0.009**
Yes	28	11.3(10.5,20.0)	1 (Reference)		39.1(27.9,65.3)	1 (Reference)	
No	199	9.5(9.0,10.4)	1.55(0.95,2.52)		25.0(20.6,26.8)	1.92(1.16,3.17)	
KRAS status				0.92			0.63
Wild-type	96	11.0(9.5,11.7)	1 (Reference)		31.1(22.7,36.3)	1 (Reference)	
Mutant	92	9.5(8.6,10.8)	0.98(0.70,1.37)		24.9(20.0,31.6)	1.08(0.78,1.51)	
RAS status				1.00			0.75
Wild-type	55	11.0(8.4,12.3)	1 (Reference)		26.3(19.8,35.8)	1 (Reference)	
Mutant	115	9.5(8.8,10.3)	1.00(0.69,1.46)		24.0(20.5,28.6)	1.06(0.73,1.53)	
BRAF status				**0.004**			**<0.001**
Wild-type	176	10.3(9.3,11.1)	1 (Reference)		27.1(23.9,33.1)	1 (Reference)	
Mutant	12	5.4(1.6,11.0)	2.40(1.28,4.50)		10.7(3.1,24.8)	2.86(1.53,5.34)	

p values < 0.05 were shown in bold. * *p* value was based on the log-rank test for PFS and OS in the univariate analysis (†).

Table S5. Subgroup analysis of *KRAS* mutant and *RAS* mutant in the discovery cohort.

	Tumor response				Progression-free survival					Overall Survival				
	N	CR+PR	SD+PD	P value*	Median, months (95%CI)	HR (95%CI)†	P value*	HR (95%CI) ‡	P value*	Median, months (95%CI)	HR (95%CI)†	P value*	HR (95%CI) ‡	P value*
***KRAS* mutant**														
GTF2H1 rs4596				0.088			0.90		0.91			0.91		0.87
G/G	24	19(79.2%)	5(20.8%)		9.5(5.4,15.2)	Reference		Reference		26.5(14.4,NE)	Reference		Reference	
G/C	58	37(67.3%)	18(32.7%)		12.0(10.4,15.0)	0.87(0.49,1.56)		0.91(0.47,1.75)		30.4(23.4,32.6)	1.13(0.64,2.00)		0.99(0.51,1.93)	
C/C	19	9(47.4%)	10(52.6%)		12.4(7.8,20.2)	0.92(0.42,2.04)		1.07(0.45,2.50)		19.6(16.2,44.3)	1.05(0.50,2.18)		1.19(0.55,2.58)	
				0.13			0.66		0.86			0.71		0.87
G/G	24	19(79.2%)	5(20.8%)		9.5(5.4,15.2)	Reference		Reference		26.5(14.4,NE)	Reference		Reference	
AnyC	77	46(62.2%)	28(37.8%)		12.0(10.9,13.1)	0.88(0.50,1.55)		0.95(0.51,1.76)		28.6(21.7,31.7)	1.11(0.64,1.93)		1.05(0.56,1.97)	
RPA rs7356				0.61			0.68		0.74			0.24		0.29
T/T	37	25(67.6%)	12(32.4%)		9.9(6.5,13.1)	Reference		Reference		21.5(17.7,31.7)	Reference		Reference	
T/C	51	30(62.5%)	18(37.5%)		12.1(9.5,14.0)	1.10(0.65,1.87)		1.11(0.63,1.94)		28.5(21.6,39.1)	0.78(0.48,1.27)		0.84(0.50,1.40)	
C/C	13	10(76.9%)	3(23.1%)		12.2(10.3,23.1)	0.76(0.31,1.87)		0.77(0.29,2.04)		36.0(18.7,NE)	0.53(0.24,1.16)		0.51(0.22,1.19)	
				0.39			0.43		0.49			0.19		0.16
AnyT	88	55(64.7%)	30(35.3%)		11.7(9.5,13.1)	Reference		Reference		26.5(20.5,30.8)	Reference		Reference	
C/C	13	10(76.9%)	3(23.1%)		12.2(10.3,23.1)	0.72(0.31,1.66)		0.72(0.29,1.80)		36.0(18.7,NE)	0.61(0.29,1.28)		0.56(0.25,1.25)	
***RAS* mutant**														
GTF2H1 rs4596				0.096			0.96		0.95			0.95		0.60
G/G	28	22(78.6%)	6(21.4%)		9.5(6.7,15.2)	Reference		Reference		25.8(17.7,41.3)	Reference		Reference	
G/C	64	42(68.9%)	19(31.1%)		12.0(10.4,15.3)	0.92(0.54,1.59)		0.93(0.51,1.72)		30.7(24.0,33.4)	1.07(0.63,1.81)		0.92(0.50,1.69)	
C/C	22	11(50.0%)	11(50.0%)		12.4(9.3,17.6)	0.96(0.47,1.95)		1.03(0.49,2.21)		21.5(16.2,44.3)	1.11(0.57,2.16)		1.26(0.63,2.53)	
				0.15			0.78		0.89			0.78		0.95
G/G	28	22(78.6%)	6(21.4%)		9.5(6.7,15.2)	Reference		Reference		25.8(17.7,41.3)	Reference		Reference	
AnyC	86	53(63.9%)	30(36.1%)		12.1(10.9,13.4)	0.93(0.55,1.57)		0.96(0.54,1.71)		28.3(22.0,31.7)	1.08(0.65,1.79)		1.02(0.58,1.80)	
RPA rs7356				0.35			0.90		0.84			0.37		0.39
T/T	44	31(70.5%)	13(29.5%)		11.0(7.7,15.6)	Reference		Reference		23.3(17.8,30.6)	Reference		Reference	
T/C	55	32(61.5%)	20(38.5%)		12.4(9.7,15.2)	1.09(0.67,1.77)		1.10(0.65,1.84)		30.2(21.6,39.1)	0.81(0.51,1.28)		0.88(0.54,1.42)	
C/C	15	12(80.0%)	3(20.0%)		12.1(9.1,23.1)	0.95(0.43,2.10)		0.88(0.37,2.09)		31.3(18.7,NE)	0.63(0.31,1.27)		0.59(0.28,1.25)	
				0.27			0.78		0.64			0.30		0.21
AnyT	99	63(65.6%)	33(34.4%)		11.7(9.7,13.4)	Reference		Reference		26.9(21.5,31.7)	Reference		Reference	

C/C	15	12(80.0%)	3(20.0%)		12.1(9.1,23.1)	0.90(0.43,1.89)		0.83(0.37,1.84)		31.3(18.7,NE)	0.70(0.36,1.36)		0.63(0.31,1.29)	

CR, complete response; PR, partial response; SD, stable disease; PD, progressive disease. *P* values<0.05 were shown in bold. * *P* value was based on the Fisher's exact test for response, log-rank test in the univariate analysis (†) and Wald test in the multivariable analysis within Cox regression model (‡) adjusted for sex, age, ECOG performance status, primary tumor site, number of metastases, primary tumor resected, adjuvant chemotherapy, RAS status, and BRAF status.

Table S6. Subgroup analysis by *KRAS* and *RAS* status in the control cohort.

	**Tumor response**				**Progression-free survival**					**Overall Survival**				
	**N**	**CR+PR**	**SD+PD**	**P value***	**Median, months (95%CI)**	**HR (95%CI)†**	**P value***	**HR (95%CI) ‡**	**P value***	**Median, months (95%CI)**	**HR (95%CI)†**	**P value***	**HR (95%CI) ‡**	**P value***
***KRAS* wild-type**														
GTF2H1 rs4596				0.45			0.44		0.51			0.71		0.24
G/G	30	17(56.7%)	13(43.3%)		11.3(8.8,15.4)	Reference		Reference		33.0(22.7,44.3)	Reference		Reference	
G/C	50	31(63.3%)	18(36.7%)		10.6(8.1,12.2)	1.35(0.79,2.31)		1.40(0.77,2.56)		31.1(20.0,39.6)	1.18(0.71,1.97)		1.66(0.89,3.08)	
C/C	15	10(76.9%)	3(23.1%)		11.1(5.8,13.1)	1.45(0.67,3.12)		1.10(0.44,2.77)		23.9(10.4,35.8)	1.32(0.64,2.72)		1.80(0.76,4.27)	
				0.38			0.21		0.32			0.45		0.092
G/G	30	17(56.7%)	13(43.3%)		11.3(8.8,15.4)	Reference		Reference		33.0(22.7,44.3)	Reference		Reference	
Any C	65	41(66.1%)	21(33.9%)		10.8(9.3,11.7)	1.37(0.82,2.30)		1.35(0.75,2.43)		26.8(20.8,37.8)	1.21(0.74,1.98)		1.68(0.92,3.07)	
RPA2 rs7356				0.42			0.80		0.60			0.75		0.69
T/T	34	22(66.7%)	11(33.3%)		10.8(7.9,12.6)	Reference		Reference		33.0(22.0,40.2)	Reference		Reference	
T/C	51	32(65.3%)	17(34.7%)		11.0(9.0,12.2)	1.18(0.71,1.97)		1.06(0.58,1.96)		32.5(21.5,39.8)	1.04(0.63,1.70)		0.81(0.47,1.39)	
C/C	11	5(45.5%)	6(54.5%)		13.1(6.7,14.7)	1.09(0.49,2.44)		0.66(0.24,1.84)		19.1(12.5,36.1)	1.32(0.62,2.85)		0.72(0.29,1.79)	
				0.19			0.99		0.33			0.46		0.69
Any T	85	54(65.9%)	28(34.1%)		11.0(9.3,11.6)	Reference		Reference		32.5(23.9,39.1)	Reference		Reference	
C/C	11	5(45.5%)	6(54.5%)		13.1(6.7,14.7)	1.00(0.47,2.10)		0.63(0.25,1.58)		19.1(12.5,36.1)	1.30(0.64,2.62)		0.85(0.38,1.91)	
***RAS* wild-type**														
GTF2H1 rs4596				0.31			0.30		0.063			0.47		0.22
G/G	16	8(50.0%)	8(50.0%)		12.6(7.7,17.2)	Reference		Reference		33.8(14.4,NE)	Reference		Reference	
G/C	29	14(50.0%)	14(50.0%)		10.5(6.7,11.7)	1.73(0.82,3.67)		2.82(1.10,7.24)		24.8(11.2,37.8)	1.52(0.74,3.14)		2.29(0.90,5.85)	
C/C	10	7(77.8%)	2(22.2%)		11.2(2.8,13.1)	1.38(0.52,3.67)		1.18(0.38,3.70)		24.4(2.8,35.8)	1.54(0.61,3.91)		2.02(0.67,6.05)	
				0.65			0.15		0.10			0.22		0.084
G/G	16	8(50.0%)	8(50.0%)		12.6(7.7,17.2)	Reference		Reference		33.8(14.4,NE)	Reference		Reference	
Any C	39	21(56.8%)	16(43.2%)		10.8(7.9,12.2)	1.63(0.79,3.36)		2.06(0.87,4.86)		24.8(14.6,35.8)	1.53(0.76,3.07)		2.20(0.90,5.38)	
RPA2 rs7356				0.48			0.61		0.16			0.92		0.24

T/T	19	10(52.6%)	9(47.4%)		9.0(4.2,11.2)	Reference		Reference		26.3(10.2,39.7)	Reference		Reference	
T/C	28	16(61.5%)	10(38.5%)		11.3(8.1,12.4)	0.85(0.43,1.70)		0.68(0.30,1.53)		27.8(15.1,48.6)	0.88(0.45,1.71)		0.53(0.24,1.13)	
C/C	8	3(37.5%)	5(62.5%)		13.1(1.8,14.7)	0.61(0.22,1.72)		0.24(0.06,1.04)		20.7(11.0,NE)	0.88(0.34,2.28)		0.50(0.16,1.58)	
				0.29			0.40		0.10			0.90		0.69
Any T	47	26(57.8%)	19(42.2%)		10.8(8.1,12.2)	Reference		Reference		26.3(20.5,35.8)	Reference		Reference	
C/C	8	3(37.5%)	5(62.5%)		13.1(1.8,14.7)	0.68(0.26,1.75)		0.33(0.09,1.25)		20.7(11.0,NE)	0.95(0.40,2.26)		0.81(0.29,2.26)	
***KRAS* mutant**														
GTF2H1 rs4596				0.97			0.87		0.67			0.70		0.21
G/G	26	14(53.8%)	12(46.2%)		9.4(6.7,11.9)	Reference		Reference		27.1(16.4,37.5)	Reference		Reference	
G/C	39	21(55.3%)	17(44.7%)		9.7(8.6,11.1)	0.89(0.50,1.58)		1.03(0.55,1.93)		23.0(19.5,43.5)	0.93(0.52,1.67)		1.34(0.71,2.51)	
C/C	26	13(52.0%)	12(48.0%)		8.7(7.5,11.9)	1.03(0.55,1.93)		1.32(0.66,2.61)		25.4(16.3,37.1)	1.18(0.64,2.20)		1.85(0.93,3.67)	
				0.99			0.82		0.67			0.93		0.16
G/G	26	14(53.8%)	12(46.2%)		9.4(6.7,11.9)	Reference		Reference		27.1(16.4,37.5)	Reference		Reference	
AnyC	65	34(54.0%)	29(46.0%)		9.5(8.6,11.1)	0.94(0.56,1.59)		1.13(0.64,2.01)		24.8(20.0,31.6)	1.03(0.60,1.75)		1.51(0.85,2.69)	
RPA rs7356				0.85			0.16		0.16			0.92		0.65
T/T	41	20(50.0%)	20(50.0%)		9.7(8.1,11.9)	Reference		Reference		25.2(18.3,37.5)	Reference		Reference	
T/C	42	23(56.1%)	18(43.9%)		9.5(8.6,11.8)	1.05(0.64,1.74)		1.01(0.58,1.75)		25.4(19.7,33.1)	0.91(0.55,1.50)		0.77(0.44,1.35)	
C/C	9	5(55.6%)	4(44.4%)		7.6(1.6,9.5)	2.06(0.93,4.56)		2.25(0.93,5.46)		18.8(5.5,NE)	1.04(0.44,2.51)		0.76(0.29,2.04)	
				0.89			0.057		0.055			0.83		0.82
AnyT	83	43(53.1%)	38(46.9%)		9.6(8.8,11.1)	Reference		Reference		25.2(20.3,32.0)	Reference		Reference	
C/C	9	5(55.6%)	4(44.4%)		7.6(1.6,9.5)	2.01(0.95,4.24)		2.24(0.98,5.10)		18.8(5.5,NE)	1.09(0.47,2.53)		0.90(0.36,2.24)	
***RAS* mutant**														
GTF2H1 rs4596				0.95			0.73		0.62			0.46		0.093
G/G	35	20(57.1%)	15(42.9%)		9.2(7.6,11.6)	Reference		Reference		26.1(19.0,35.1)	Reference		Reference	
G/C	50	29(59.2%)	20(40.8%)		9.7(8.6,11.6)	0.90(0.55,1.46)		0.98(0.57,1.69)		24.5(19.8,39.8)	0.86(0.52,1.41)		1.08(0.63,1.86)	
C/C	29	15(55.6%)	12(44.4%)		8.7(7.3,11.8)	1.10(0.63,1.93)		1.27(0.68,2.37)		23.9(14.1,32.0)	1.19(0.69,2.05)		1.85(1.01,3.39)	
				0.94			0.87		0.81			0.88		0.32
G/G	35	20(57.1%)	15(42.9%)		9.2(7.6,11.6)	Reference		Reference		26.1(19.0,35.1)	Reference		Reference	
AnyC	79	44(57.9%)	32(42.1%)		9.5(8.6,11.1)	0.96(0.61,1.51)		1.07(0.64,1.78)		24.0(20.0,29.1)	0.96(0.62,1.51)		1.29(0.78,2.12)	
				0.80			0.51		0.33			0.28		**0.031**
Any G	85	49(58.3%)	35(41.7%)		9.6(8.8,11.1)	Reference		Reference		25.0(20.6,33.1)	Reference		Reference	
C/C	29	15(55.6%)	12(44.4%)		8.7(7.3,11.8)	1.18(0.73,1.91)		1.29(0.77,2.15)		23.9(14.1,32.0)	1.30(0.81,2.08)		1.76(1.05,2.93)	
RPA rs7356				0.99			0.078		0.11			0.83		1.00
T/T	50	27(56.3%)	21(43.8%)		10.3(8.1,11.9)	Reference		Reference		25.4(19.8,37.5)	Reference		Reference	
T/C	53	30(57.7%)	22(42.3%)		9.5(8.6,11.5)	1.18(0.75,1.84)		1.12(0.70,1.79)		24.0(19.7,32.5)	1.00(0.64,1.55)		0.98(0.61,1.57)	
C/C	12	7(58.3%)	5(41.7%)		7.6(3.6,9.5)	2.12(1.07,4.22)		2.29(1.05,4.99)		18.9(9.8,NE)	1.24(0.60,2.56)		0.99(0.44,2.23)	

				0.93			**0.032**		**0.040**			0.54		0.99
AnyT	103	57(57.0%)	43(43.0%)		9.6(8.8,11.1)	Reference		Reference		25.0(20.8,32.0)	Reference		Reference	
C/C	12	7(58.3%)	5(41.7%)		7.6(3.6,9.5)	1.95(1.03,3.72)		2.15(1.03,4.46)		18.9(9.8,NE)	1.24(0.62,2.47)		1.00(0.47,2.14)	

CR, complete response; PR, partial response; SD, stable disease; PD, progressive disease. *P* values<0.05 were shown in bold. * *P* value was based on the Fisher's exact test for response, log-rank test in the univariate analysis (†) and Wald test in the multivariable analysis within Cox regression model (‡) adjusted for sex, age, ECOG performance status, primary tumor site, number of metastases, primary tumor resected, adjuvant chemotherapy, RAS status, and BRAF status.
